# Degradation shaped bacterial and archaeal communities with predictable taxa and their association patterns in Zoige wetland at Tibet plateau

**DOI:** 10.1038/s41598-018-21874-0

**Published:** 2018-03-01

**Authors:** Yunfu Gu, Yan Bai, Quanju Xiang, Xiumei Yu, Ke Zhao, Xiaoping Zhang, Chaonan Li, Songqing Liu, Qiang Chen

**Affiliations:** 1 Department of Microbiology, College of Resource Sciences and Technology, Sichuan Agricultural University, Chengdu, 611130 China; 2 Environmental Microbiology Key Laboratory of Sichuan Province, Chengdu Institute of Biology, Chinese Academy of Sciences, Chengdu, 610041 China; 3 Department of Microbiology, College of Chemistry and Life Science, Chengdu Normal University, Chengdu, 611130 China

## Abstract

Soil microbes provide important ecosystem services. Zoige Plateau wetland, the largest alpine peat wetland in the world, has suffered from serious degradation in the past 30 years. We studied the composition of the Zoige Plateau alpine wetland soil microbiota and relations among specific taxa using 16S rRNA amplicon sequencing combined with association network analysis. Compared to the pristine swamp soil, taxons DA101, *Aeromicrobium*, *Bradyrhizobium*, and *Candidatus Nitrososphaera* were enriched and several methanogenic Euryarchaeota were depleted in the moderately degraded meadow soil and highly degraded sandy soil. Soil total potassium contents in soils with different degradation levels were significantly different, being the highest in meadow soil and lowest in swamp soil. The association network analysis showed that total potassium positively correlated with specific bacterial and archaeal taxa. *Jiangella*, *Anaerolinea*, *Desulfobulbus*, *Geobacter*, *Flavobacterium*, *Methanobacterium* and *Methanosaeta* were identified as the keystone genera in the networks. Soil degradation affected soil properties, and caused changes in the bacterial and archaeal community composition and the association patterns of community members. The changes could serve as early warning signals of soil degradation in alpine wetlands.

## Introduction

Despite the increased understanding of the important roles of wetlands in terrestrial ecosystems, severe land loss and degradation occur in the wetlands of China due to population growth, land reclamation projects and over-grazing^1^. The alpine wetlands are more vulnerable to climate change than those in other regions^2^. The Zoige alpine wetland on the Qinghai-Tibetan Plateau is the highest and largest alpine wetland in the world. Zoige wetland plays important roles in maintaining water balance, carbon sequestration, and biodiversity preservation^3^. It covers an area of 1.2 × 10^4^ km^2^, occupying about 0.1% of the total global peatland area^4^. In the past 40 years, numerous studies have focused on the degradation of Zoige wetland that has resulted from increasing temperature, anthropogenic perturbations, and overgrazing by cattle. The studies have shown significant impacts of these activities on the landscape, hydrology, biodiversity, and ecosystem degradation, including severe peat deterioration. The mean annual temperature has increased at a rate of 0.16 °C per decade in this area, resulting in the drying of the wetlands^5^. The wetland area in Zoige has decreased by approximately 30% from the 1970s to the 2000s due to the decline of water table^6^, resulting in a transition from pristine swamp soil to degraded meadow soil, and even aeolian sandy soil at severely deteriorated sites^7,8^. Deterioration has also changed the composition of plant communities from humidogenes and aquatics to mesophytes and xerophytes^9^, and resulted in the decomposition of soil organic matter, loss of humus and peat horizon, and decrease in nutrient content^10,11^.

Soil microbes are critical in biogeochemical processes and organic material decomposition, and quickly respond to environmental disturbances^12,13^. At high taxonomic ranks, for example at phylum or class level, bacteria and archaea exhibit ecological coherence, and community composition may be valuable in predicting ecosystem functioning^14^. Based on nutritional requirements and growth rates, microbes can be classified as either copiotrophs or oligotrophs at the phylum level, and applying this classification may contribute to further understanding of microbial community dynamics in response to environmental interferences^15^. Alpine wetland degradation could directly alter the composition of specific bacterial and archaeal phyla. For example, *Bacteroidetes* and *Flavobacterium* were widely distributed in the pristine swamp soil in Zoige wetland, while *Alphaproteobacteria* were more dominant in the degraded sandy soil^16^. However, knowledge on the response of bacterial and archaeal taxa at low taxonomical level (such as genus or species) to the alpine wetland degradation remains limited. It is likely that the loss of humus due to wetland degradation would select for specific taxa that depend primarily on organic substrates, resulting in changes in microbial community composition and soil nutrient status^17,18^. Taxa that are affected by alpine wetland degradation should be associated with edaphic properties like soil organic carbon, which could then be used as valuable indicators of alpine wetland soil nutrient status and development^16^. Microbial communities in soil are complex, including many potentially interacting taxa, and a full exploration of these interactions is important to allow an integrated understanding of soil microbial community structure and the ecological rules that govern them^19^. Co-abundance network analyses can be carried out using data generated from high-throughput sequencing to provide information on the community composition and interactions within communities^19^. Furthermore, co-presence links reflect shared niches while exclusion links suggest niche segregation.

Knowledge on the effects of wetland degradation on bacterial and archaeal communities is still scarce. To address this, we applied 16S rRNA gene amplicon sequencing to detect microbial community variation in microhabitats associated with the succession of degradation, and determined factors associated with variation. The effects of wetland degradation on specific bacterial and archaeal taxa and their associations with soil nutrients were analyzed by analyzing differences in abundant taxa and by co-abundance network analysis. Our purpose was to determine (1) the variation in bacterial and archaeal communities, (2) the specific taxa affected by wetland degradation in the three soils, and (3) the possible relationships between specific taxa and soil properties. We hypothesized that (i) the bacterial and archaeal communities in the pristine soil would significantly differ with those found in the degraded soil due to the inherent differences in nutritional conditions, (ii) that taxa related to carbon and nitrogen cycling would either be enriched/or depleted, and (iii) the specific taxa that were substantially changed would form distinct association patterns.

## Results

### Soil properties and community composition

**Figure 1:**
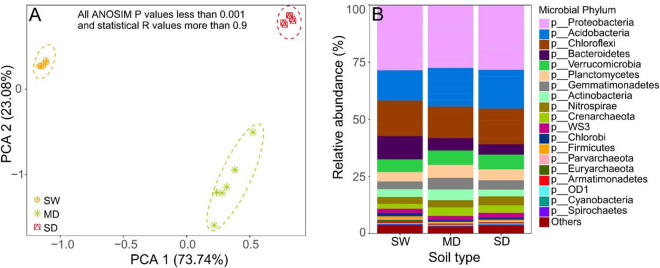
Principal component analysis (PCA) of soil edaphic properties (**A**) and microbial community composition in Zoige plateau wetland soils (**B**). SW: swamp soil; MD: meadow soil; and SD: sandy soil.

Soil properties were different in the three soils associated with the degraded Zoige wetland (Table 1). Soil total potassium content (TK: 6.79 to 23.13 g kg^−1^) was highest in the meadow soil and lowest in swamp soil (Table 1). Principal component analysis (PCA) showed clear separation of the three soils indicating that wetland degradation resulted in distinct microhabitats (ANOSIM: R > 0.9, *P* < 0.001) (Fig. 1A). *Proteobacteria* (24.6–31.6%), *Acidobacteria* (12.3–19.7%), and *Chloroflexi* (10.8–19.7%) were the dominant phyla in all soil types. In the swamp soil, the relative abundance of *Proteobacteria* was highest, while that of *Acidobacteria* was lower than in the other soils. *Chloroflexi* was more abundant in the swamp and sandy soils than in the meadow (Fig. 1B). Within *Proteobacteria*, the *Delta* subdivision was the most abundant group (34.7 to 40.0%), followed by *Betaproteobacteria* (26.9 to 29.4%), *Alphaproteobacteria* (16.5–22.6%), and *Gammaproteobacteria* (14.0–15.4%) (Fig. S1). The relative abundances of *Deltaproteobacteria* and *Betaproteobacteria* were higher in the swamp soil than in the other soils. The relative abundance of *Alphaproteobacteria* was highest in the swamp soil, and that of *Gammaproteobacteria* in the sandy soil. The relative abundances of ten families were higher than 0.5% of the total abundance (Fig. S2). Compared to the pristine swamp soil, the relative abundance of *Nitrososphaeraceae*, *Sphingomonadaceae*, *Xanthomonadaceae*, and *Chitinophagaceae* were significantly higher in the degraded soils, while those of *Sinobacteraceae* and *Comamonadaceae* were lower (P < 0.05). Moreover, the relative abundances of *Myxococcaceae* (P < 0.05) and *Rhodocyclaceae* were lowest in the meadow soil, while those of *Syntrophobacteraceae* and *Rhodospirillaceae* were lowest in the swamp soil (Fig. S2).

**Table 1:** Physicochemical parameters of soil samples from Zoige plateau wetland.

Soil codes*	WC (%)	pH	SOC (g.kg^−1^)	TN (g.kg^−1^)	OP (g.kg^−1^)	TK (g.kg^−1^)	AN (mg.kg^−1^)	AP (mg.kg^−1^)	AK (mg.kg^−1^)
SW11	74.9 ± 0.81a	7.59 ± 0.09b	279.1 ± 5.69a	5.15 ± 1.16a	1.43 ± 0.09a	7.44 ± 0.05c	993.0 ± 28.5a	26.0 ± 3.08a	99.8 ± 2.58b
SW13	73.6 ± 1.49a	7.58 ± 0.12b	283.4 ± 10.9a	4.99 ± 0.18a	1.45 ± 0.11a	7.46 ± 0.11c	1045.3 ± 38.9a	24.1 ± 0.91a	97.5 ± 1.00b
SW22	74.3 ± 0.65a	7.61 ± 0.01b	278.7 ± 4.62a	5.45 ± 0.38a	1.38 ± 0.08a	7.13 ± 0.43c	1038.2 ± 47.4a	25.1 ± 0.68a	101.2 ± 1.12b
SW23	76.5 ± 1.70a	7.66 ± 0.04b	283.1 ± 3.74a	5.71 ± 0.09a	1.41 ± 0.02a	6.79 ± 0.15c	1079.4 ± 91.2a	22.6 ± 0.21a	96.7 ± 0.54b
SW32	79.4 ± 2.55a	7.56 ± 0.05b	281.3 ± 1.10a	5.29 ± 0.03a	1.32 ± 0.03a	7.30 ± 0.05c	1025.9 ± 8.29a	24.7 ± 0.55a	98.3 ± 5.13b
SW33	83.1 ± 2.35a	7.62 ± 0.10b	281.4 ± 18.3a	5.71 ± 0.29a	1.33 ± 0.02a	6.94 ± 0.02c	1014.8 ± 1.83a	24.1 ± 1.39a	96.2 ± 1.42b
MD7	16.6 ± 1.48b	7.98 ± 0.08a	72.8 ± 1.83b	3.67 ± 0.18b	0.95 ± 0.01b	22.3 ± 0.06a	277.4 ± 17.7b	11.2 ± 0.82b	443.5 ± 40.5a
MD15	16.1 ± 0.91b	7.84 ± 0.09a	66.3 ± 4.88b	3.22 ± 0.06b	0.98 ± 0.19b	21.3 ± 0.07a	246.3 ± 3.52b	12.4 ± 2.04b	365.4 ± 4.67a
MD78	14.6 ± 0.86b	7.75 ± 0.04a	55.7 ± 2.75b	2.99 ± 0.10b	0.44 ± 0.08b	23.1 ± 0.51a	195.3 ± 3.77b	11.9 ± 0.94b	103.1 ± 4.66a
MD62	17.3 ± 0.90b	7.83 ± 0.03a	65.9 ± 5.70b	3.12 ± 0.17b	0.68 ± 0.02b	22.4 ± 1.38a	199.4 ± 6.30b	12.2 ± 0.52b	253.7 ± 34.7a
MD35	15.2 ± 0.65b	7.86 ± 0.05a	69.1 ± 1.07b	3.08 ± 0.11b	0.88 ± 0.06b	21.9 ± 0.15a	221.8 ± 1.67b	10.9 ± 0.50b	333.1 ± 34.7a
MD14	14.8 ± 0.65b	7.88 ± 0.09a	59.9 ± 0.76b	3.68 ± 0.07b	0.81 ± 0.03b	22.5 ± 1.17a	297.9 ± 7.31b	12.4 ± 1.04b	325.2 ± 67.5a
SD67	7.13 ± 0.46c	6.74 ± 0.36c	7.99 ± 0.13c	0.81 ± 0.04c	0.91 ± 0.05b	17.8 ± 0.69b	45.3 ± 0.53c	4.29 ± 0.41c	47.5 ± 1.39b
SD68	6.89 ± 0.59c	6.71 ± 0.25c	8.02 ± 0.22c	0.79 ± 0.14c	0.72 ± 0.02b	16.9 ± 0.13b	41.2 ± 5.52c	3.78 ± 0.27c	41.3 ± 0.61b
SD70	7.01 ± 0.08c	6.71 ± 0.03c	8.24 ± 0.12c	0.81 ± 0.11c	0.76 ± 0.01b	16.7 ± 0.34b	61.2 ± 3.51c	3.45 ± 0.19c	33.4 ± 1.97b
SD73	8.11 ± 0.12c	6.73 ± 0.08c	8.04 ± 0.11c	0.83 ± 0.05c	0.65 ± 0.04b	16.7 ± 0.17b	60.5 ± 5.69c	3.69 ± 0.10c	42.77 ± 3.74b
SD75	5.89 ± 0.65c	6.69 ± 0.08c	8.78 ± 0.49c	0.82 ± 0.03c	0.76 ± 0.02b	16.8 ± 0.20b	62.1 ± 2.30c	3.77 ± 0.32c	35.7 ± 0.12b
SD76	7.23 ± 0.05c	6.74 ± 0.10c	8.55 ± 0.19c	0.86 ± 0.05c	0.84 ± 0.04b	17.7 ± 0.16b	65.4 ± 8.26c	4.52 ± 0.07c	51.2 ± 0.94b

Mean ± standard error (n = 3). Values within the same column followed by the same letter do not differ at *P* < 0.05. WC: water content; SOC: soil organic carbon; TN: total nitrogen; TP: total phosphorus; TK: total potassium; AN: available nitrogen; OP: Olsen phosphorus (available phosphorus); AK: available potassium. *SW: swamp soil; MD: meadow soil; SD: sandy soil.

### Differentially abundant taxa

**Figure 2:**
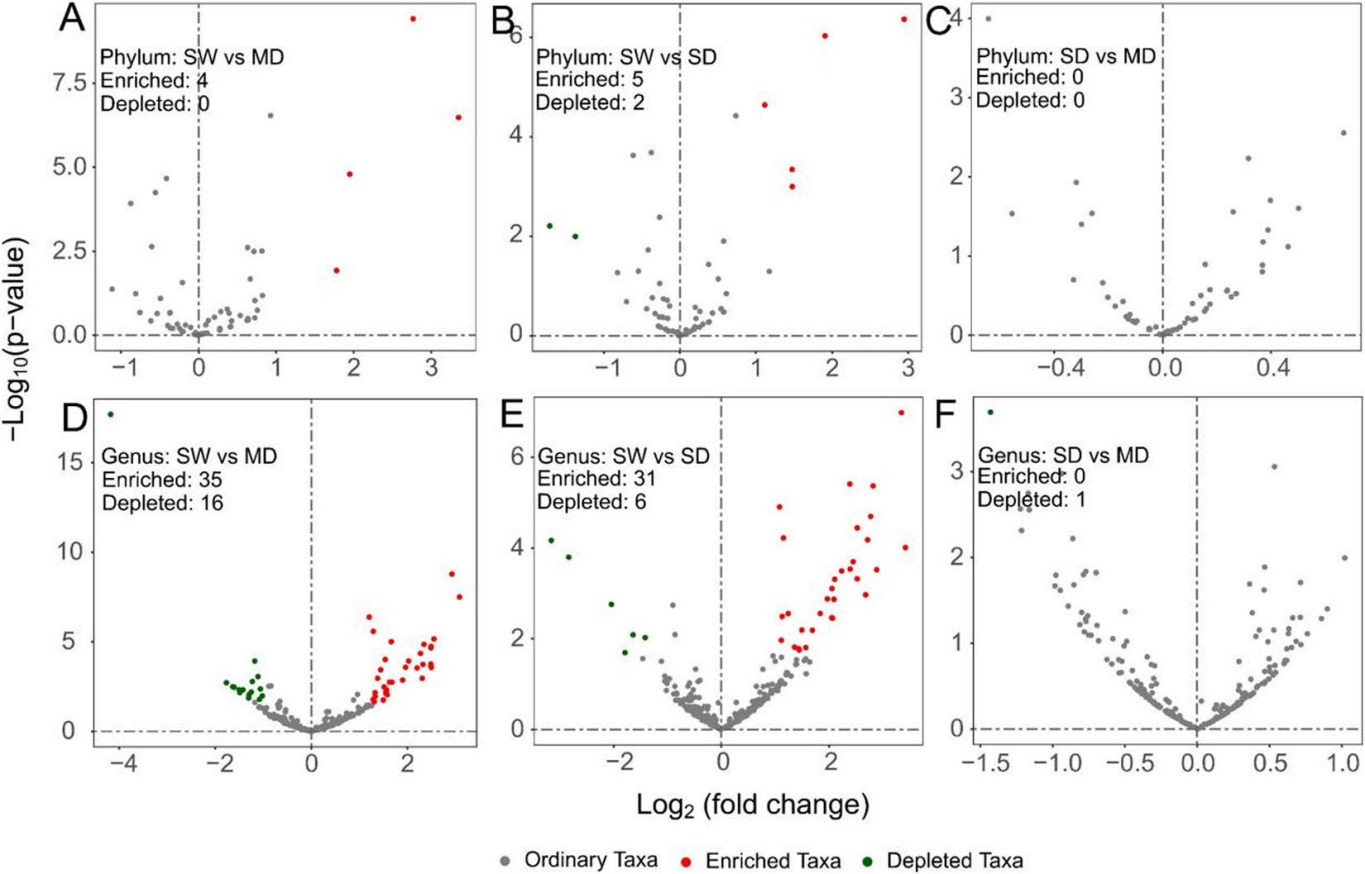
Volcano plots illustrating taxa that were significantly enriched (red) and depleted (green) at phylum (**A,B** and **C**) and genus level (**D**,**E** and **F**). SW: swamp soil; MD: meadow soil; and SD: sandy soil.

Enriched taxa (e-taxa) and depleted taxa (d-taxa), representing taxa with relative abundances twice higher or lower (P = 0.05) in the swamp soil than in the meadow and sandy soils, were identified by differential abundance analysis. Using taxa counts from swamp soil as a control and an FDR-adjusted P < 0.1, eleven and 89 differentially abundant taxa were detected at the phylum and genus levels, respectively (Fig. 2).

At the phylum level, *Euryarchaeota*, WWE1, OP8 and GOUTA4 were enriched in the swamp soil when compared with the meadow soil (Figs 2A and S3A). Compared with the sandy soil, WWE1, *Euryarchaeota*, OP8, GOUTA4 and *Bacteroidetes* were enriched, and TM7 and AD3 were depleted in the swamp soil (Figs 2B and S3B). At the genus level, additional 35 e-taxa and 16 d-taxa were detected in the swamp soil when compared with the meadow soil (Fig. 2D). The top five e-taxa were DA101, *Crocinitomix*, *Aeromicrobium*, *Bradyrhizobium*, and *Candidatus Nitrososphaera* (phylum *Crenarchaeota*), and the top five d-taxa were *Syntrophus*, *Methanobacterium*, *Methanosarcina*, *Chloronema*, and *Methanosaeta* (Dataset S1). When compared with the sandy soil, 31 e-taxa and 6 d-taxa were detected in the swamp soil (Fig. 2E), among which DA101, *Aeromicrobium*, *Sporichthya*, JG37-AG-70, and *Zoogloea* were the top five e-taxa and *Chryseobacterium*, *Syntrophus*, *Chloronema*, *Oryzihumus*, and *Methanosarcina* the top five d-taxa (Dataset S2). When comparing sandy soil with the meadow soil, only one d-taxa was detected at the genus level (Fig. 2C,F).

The Mantel test showed significant correlations between differentially abundant phyla and multiple edaphic factors. Soil TK correlated negatively with most of the differentially abundant phyla (P = 0.001) (Fig. S3C,D).

### Community structure, variation, and determinants

**Figure 3:**
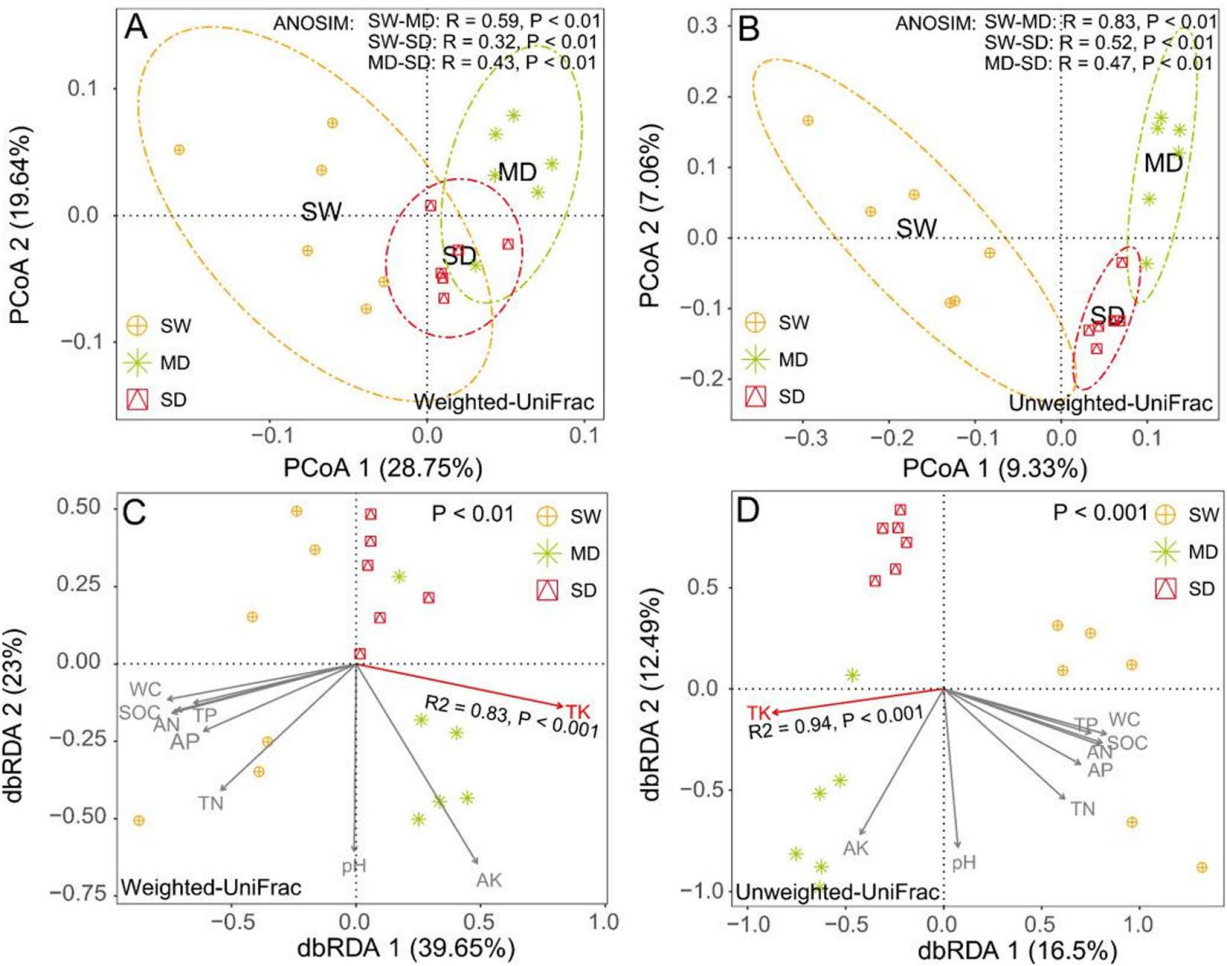
Microbial community variation across the Zoige plateau wetland soils. (**A,B**) Principal coordinate analysis (PCoA) plots of OTU-based weighted (**A**) and unweighted (**B**) UniFrac distances between samples; (**C**,**D**) distance-based redundancy analysis (dbRDA) of weighted (**C**) and unweighted (**D**) UniFrac distances quantifying the impacts of edaphic factors on microbial community composition. SW: swamp soil; MD: meadow soil; and SD: sandy soil. WC: water content; SOC: soil organic carbon; TN: total nitrogen; TP: total phosphorus; TK: total potassium; AN: available nitrogen; OP: Olsen phosphorus (available phosphorus); AK: available potassium. PDA: Potential denitrifying activity. *SW: swamp soil; MD: meadow soil; SD: sandy soil.

Principal coordinate analysis (PCoA) with weighted and unweighted UniFrac distances clearly separated the swamp communities from the communities in degraded soils (Fig. 3A,B). Weighted UniFrac takes the abundance of taxa into account whereas unweighted UniFrac is based on the presence and absence of species, making it more sensitive to rare taxa (Fig. 3B). The clear separation between the swamp soil and the degraded soils in unweighted Unifrac-based PCoA indicates that wetland degradation affects especially the rare taxa. In the distance-based redundancy analysis (dbRDA), soil TK was the strongest driver of microbial community structure among the constrained nine edaphic factors (Fig. 3C,D). In addition, Mantel test showed that soil TK correlated positively and significantly with soil microbial community composition (Fig. S4A,B). These results further suggest that the total potassium content of the soils was the key factor in shaping the microbial community composition in the wetland soils.

### Network associations

**Figure 4:**
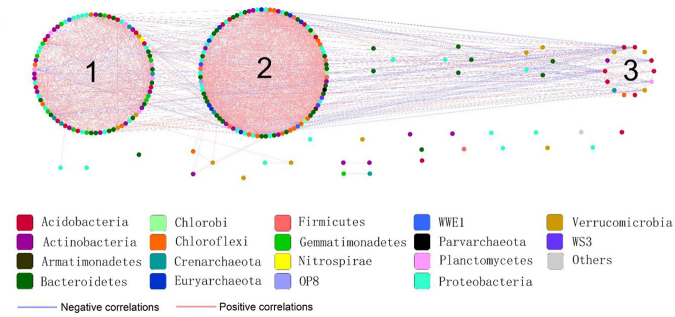
The abundance association network of differentially abundant taxa in the Zoige plateau wetland soils. Purple and red lines represent negative and positive correlation, respectively.

**Figure 5:**
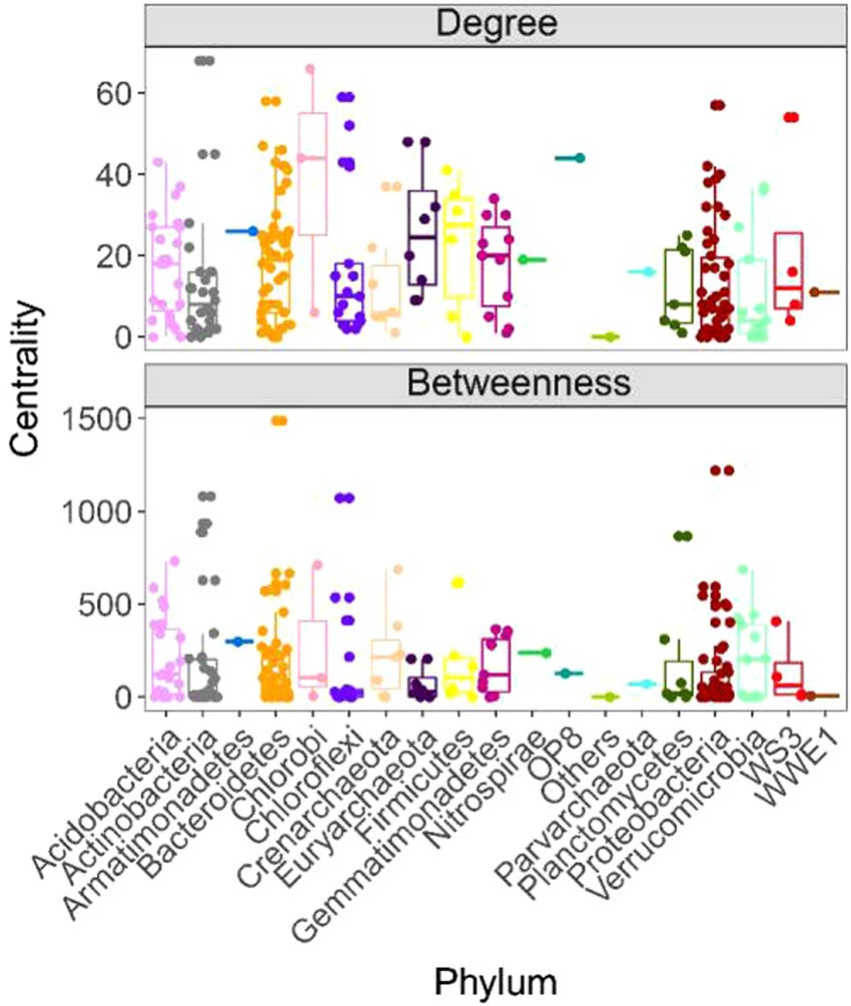
Network level topological features in the Zoige plateau wetland soils.

**Figure 6:**
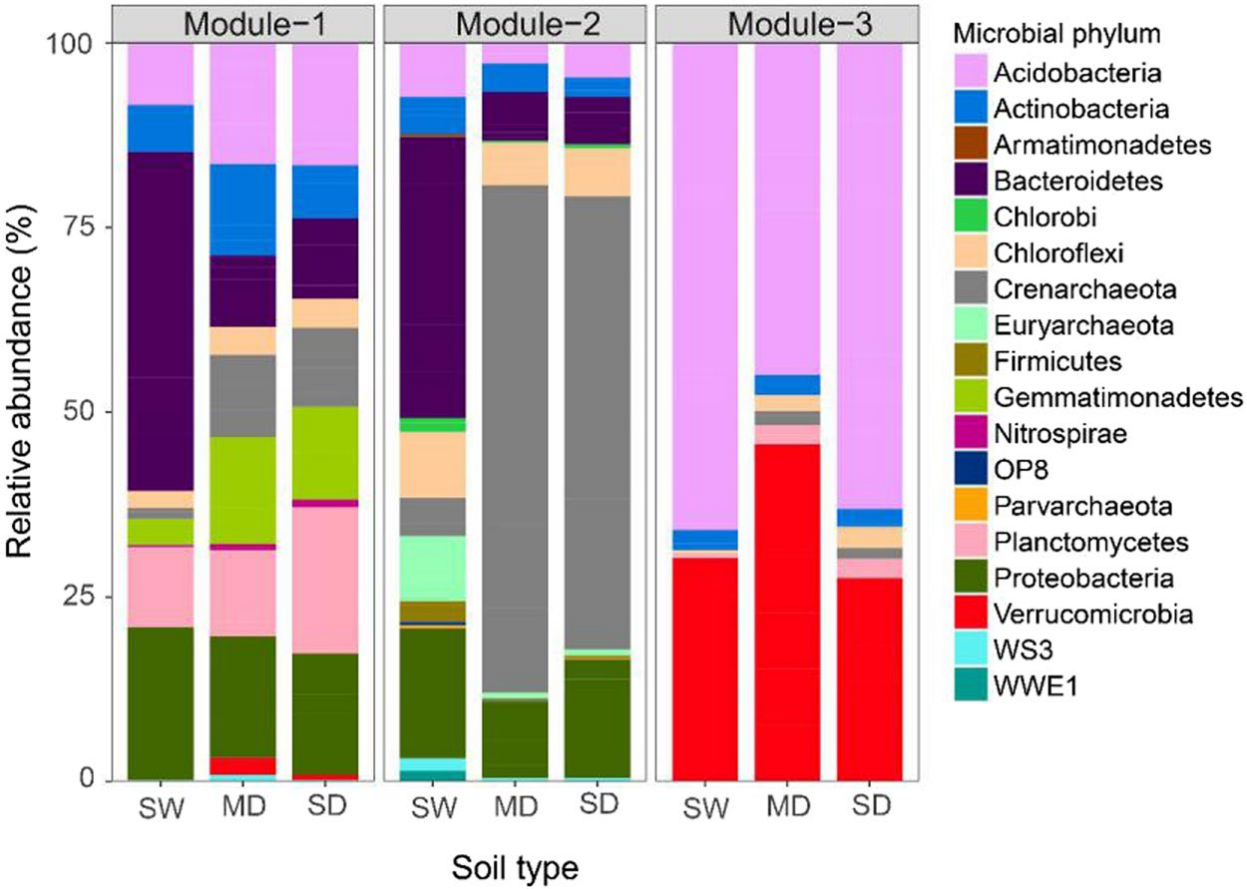
The taxonomic composition at the phylum level of the network modules in the Zoige platerau wetland soils.

Based on differential taxa abundance analysis, association networks were generated to characterize bacterial and archaeal communities in the soils (Fig. 4). The topological features of the network are presented in Table S2. In total, 1893 edges connected the 229 differentially abundant taxa in the network. Many of the differentially abundant taxa correlated with each other positively (Fig. 4), indicating that these taxa likely respond similarly to changes associated with wetland degradation. *Actinobacteria, Bacteroidetes, Chloroflexi, Euryarchaeota* and *Proteobacteria* showed higher degree centrality in the network, suggesting that these taxa are strongly associated with the other members of the community (Fig. 5). Taxa belonging to *Actinobacteria, Bacteroidetes, Chloroflexi, Proteobacteria* and *Planctomycetes* had higher betweenness centrality compared to the other taxa, indicating that these taxa likely influence the interactions among taxa in the community (Fig. 6).

Based on greedy modularity optimization, three potential functional modules (M1, M2, and M3) were detected according to association strength among the differentially abundant taxa (Fig. 4). Module M1 included *Jiangella* (phylum *Actinobacteria*), *Anaerolinea* (phylum *Chloroflexi)*, *Desulfobulbus* (*Proteobacteria)*, *Flavobacterium* (*Bacteroidetes)*, and *Methanobacterium* and *Methanosaeta* (phylum *Euryarchaeota)*. The abundances of these taxa were significantly higher in the swamp soil than in the degraded soils (Fig. 6, Table 2). With the exception of DA101 in M2 and *Acidovorax* in M3, most of the differentially abundant taxa in modules M2 and M3 were not identified at genus level. The relative abundances of almost all taxa in each module were distinct in different soils (Fig. 6). The variation in abundance and taxonomic composition may provide clues on the functional modules that potentially rapidly and reversibly respond to environmental changes induced by wetland degradation through species sorting (plastic adjustment). Although the microhabitats in the meadow and sandy soils were distinct, the composition of the functional modules was similar, especially in modules M1 and M2 (Fig. 6).

**Table 2:** Abundances of selected genera detected in the network module M1 of the three different soils associated with Zoige plateau wetland degradation.

Genus	Soils*		
	SW	MD	SD
*Jiangella*	4.33 ± 1.12a	0.00 ± 0.00b	0.00 ± 0.00b
*Anaerolinea*	69.8 ± 8.30a	29.0 ± 11.8b	31.7 ± 12.9b
*Desulfobulbus*	21.7 ± 5.67a	6.83 ± 2.79b	9.67 ± 3.95b
*Geobacter*	103.7 ± 15.4a	40.5 ± 16.5b	44.7 ± 18.2b
*Flavobacterium*	100.5 ± 45.6a	12.3 ± 5.04b	12.8 ± 5.24b
*Methanobacterium*	67.5 ± 21.3a	5.50 ± 2.25b	10.2 ± 4.15b
*Methanosaeta*	13.2 ± 5.09a	0.50 ± 0.20b	1.00 ± 0.41b

Mean ± standard error (n = 6). Values followed by the same letter within a row are not significantly different at P = 0.05. *See Table 1 for the soil code.

PCoA result showed significant (p = 0.001) correlations between network modules and soil properties, with soil TK ranking highest (Fig. S5A–C). This was supported by Mantel test, which showed high correlations between soil TK and these three potential functional modules, further indicating that soil TK drives structuring in these functional modules (Fig. S5D).

## Discussion

We studied shifts in bacterial and archaeal community composition in three soil types associated with wetland degradation: pristine swamp soil, moderately degraded meadow soil and highly degraded sandy soil. As in previous studies based on denaturing gradient gel electrophoresis^16,20^, the bacterial and archaeal community compositions in the three soils differed significantly, but all were dominated by the phyla *Proteobacteria*, *Acidobacteria* and *Chloroflexi*. *Proteobacteria* are prevalent in various ecosystems and involved in many biogeochemical processes^21^. As in Röske*et al*.^22^, *Proteobacterial* communities were mainly composed of *Deltaproteobacteria* and *Betaproteobacteria*, both classified as copiotrophs stimulated by increased nutrients in the environment^15^, which could partially explain why these two groups were more abundant in the swamp soil with higher organic matter content. *Acidobacteria* include many oligotrophs^23,24^, and their relative abundance was higher in the degraded soils that contained less organic matter, thus possibly favoring these oligotrophs. Interestingly, *Chloroflexi* that are known to survive on plant residues and grow even under nutrient limitation^25,26^ were more abundant in the sandy soil which had less nutrients than the pristine swamp soil.

Understanding the environmental variables that shape soil microbial community structuring is one of the key goals of microbial ecology. Soil water content was previously found to be the major factor connected with Zoige wetland degradation, with significant impacts on the decrease of fungal community abundance and prokaryotic diversity^20,27^. With the lowering of water table, some of the wetland plant species that are essential in swamp soil formation are being replaced by meadow vegetation^28^. Different vegetation types may release different types of root exudates: the exudates are significant sources of carbon, nitrogen and energy of the soil microbiota^29^. Different plant species also have diverse nutritional needs, promoting the development of particular soil microbiome depending on the growth substrate^30^. In our study, water content, organic carbon content, and the total and available nitrogen and phosphorus contents all decreased along with the wetland degradation, making them valuable indicators for evaluating wetland degradation. Soil total potassium content was found to be an important factor shaping the bacterial and archaeal communities. Similarly, Pereira *et al*.^31^ found that the bacterial diversity and community composition in the soil were highly affected by the total potassium content. Potassium is easily leached because of its mobility in soils, and thus directly affected by soil degradation.

The microbial community structure in the pristine swamp soil differed from those in the meadow and sandy soils. Consistent with our hypothesis, more taxa were enriched in the swamp soil, indicating that wetland degradation decreased the abundance of these bacterial and archaeal taxa. Methanogenic *Euryarchaeota* is mainly distributed in anoxic environments, for example in wetlands, paddy soils and marine sediments, and is involved in greenhouse gas production^32^. In this study, as expected, the abundances of *Euryarchaeota* were significantly higher in the pristine soils than in the degraded soils. Previous studies indicated that Zoige wetland was one of the hotspots of methane emissions in the Qinghai-Tibetan plateau, harboring a unique methanogen community^33^. In contrast, there were hardly any methanogens in the meadow soil in Zoige wetland^34^, probably due to the decreased soil water table and content that had lead to less anaerobic conditions. Although we did not measure the oxygen content in the soils, soil water content in the degraded soils had decreased significantly and water table was lower. A change in water table and content could directly affect the oxic–anoxic interfaces, increase the thickness of the boreal peatland aerobic layer, and ultimately change the microbial community composition and abundances^35^. Moreover, low water tables affect soil organic carbon (SOC) by changing the vegetation cover and SOC decomposition rate which is positively correlated with the methanogenic archaea community^36^. At the genus level, the enrichment of *Methanobacterium*, *Methanosarcina* and *Methanosaeta* in the swamp soil indicate their potential roles as ‘keystone species’ in methanogenesis in this ecosystem.

Syntrophy, defined as a long-term stable relation of organisms which can be either beneficial or unbeneficial, plays a key role in many anaerobic ecosystems, even in systems where metabolism is sustained by the reduction of electron acceptors such as sulphate or nitrogen oxides^37^. Species related to Syntrophobacteraceae generally constitute consortia with methanogenic Archaea to syntrophically convert propionate to methane, which is a typical metabolic process during anaerobic degradation. It is likely that the relative abundance of Syntrophobacteraceae affiliated species in this study would similarly vary with the Methanogenic Euryarchaeota in response to wetland degradation. However, phylogenetic analysis of the community showed that contrary to the decreased abundance of the methanogenic Euryarchaeota, the abundances of members of the Syntrophobacteraceae actually increased. Syntrophobacteraceae are extremely diverse in their nutritional transformation capabilities, with some members being able to use a wide variety of electron donors and carbon sources syntrophically or in pure cultures, and in the presence or absence of sulfate as electron acceptor^38^. This metabolic peculiarity confers these microorganisms with adaptability to changing environmental conditions, and may explain the increase of *Syntrophobacter*-related species in the degraded meadow and sandy microcosms.

DA101 can occupy various ecological niches and perform oligotrophic life strategy in soil^39^. *Aeromicrobium* are lignocellulose decomposers and play important roles in organic matter turnover in soil^40^. *Bradyrhizobium* are beneficial for plant growth due to their nitrogen-fixing ability. *Candidatus Nitrososphaera* thrives in organic-rich habitats^41^. The enrichment of these taxa in the degraded meadow and sandy soils suggest their subtle differences in habitat adaption, which also contributes differently to the carbon and nitrogen cycling in this specific ecosystem.

Network analysis provides understanding of potential interactions such as cooperation, competition and niche partitioning in bacterial and archaeal communities, and may identify keystone populations^42^. A node with high betweenness centrality value is in a core location in the network, and may affect other interactions in the community^43^. Keystone species with maximum betweenness centrality values are likely to serve as gatekeepers in ecosystem functions^44,45^. In our study, most archaeal keystone species in the networks were affiliated with *Euryarchaeota* that participate in wetland carbon cycling as discussed above, while most bacterial keystone species were from *Actinobacteria*, *Proteobacteria* and *Bacteroidetes*. Of all the keystone species detected in our study, *Desulfobulbus* are the only ones involved in the sulfur biogeochemical cycle, particularly in the reduction of sulfate to sulfide^46^. *Geobacter* are able to oxidize recalcitrant organic carbon^47^, and *Flavobacterium* are stimulated in organic matter rich environments^48^. The organic matter content in the swamp soil was higher than in the degraded soils, which may explain the significantly higher abundance of *Flavobacterium* in the swamp soil. Taken altogether, the keystone species may be pivotal determinants of the succession of bacterial and archaeal taxa during the wetland degradation.

In summary, wetland degradation changed soil properties, which further affected specific bacterial and archaeal taxa involved in nutrient cycling resulting in distinct association patterns. *Proteobacteria*, *Acidobacteria*, and *Chloroflexi* were the most abundant phyla. Soil total potassium strongly affected the shaping of bacterial and archaeal communities. Interpretation of the interaction networks in soil bacterial and archaeal community may open new avenues for understanding the ecological roles of bacterial and archaeal taxa in the degradation of the Zoige alpine wetland soil. The observed changes may serve as early warning signals of soil degradation in alpine wetlands.

## Materials and Methods

### Study area and soil sampling

The Zoige Plateau is in the northeast of Qinghai-Tibetan Plateau (101°30′E–103°30′E, 32°20′N–34°00′N) at an average altitude of 3500 m (Table S1). The mean annual precipitation in this area is 654 mm^49^. Eighty-five percent of precipitation is delivered from April to September. The mean annual air temperature is 1.1 °C^33^, with the average temperatures ranging from −10.1 °C in January to 10.7 °C in July.

Swamp soil (SW), meadow soil (MD) and sandy soil (SD) in Zoige were sampled in August 2014. The swamp was covered with water with an annual average water table of 5.87 cm above ground. The vegetation was dominated by hydrophytes and sedge hydro-mesophytes (*Carexmuliensis*, *Carexlasiocarpa* and *Carexmeyeriana*). The meadow surface was in a humid state and the vegetation was dominated by mesophytes and hydro-mesophytes (*Kobresiatibetica*), and the water table was approximately 4.26 cm below ground. The sandy soil surface was continuously dry and had little or only few *Psammophytes* as vegetation cover^50^, and the water table was at least 21 cm below ground. The soil texture was peat soil in SW, sandy loam in soil MD, and sandy soil in SD. The parent materials of swamp and meadow soils were homogeneous silt clay and Triassic slate residues, and sandstones and siltstone, respectively^51^. In the sandy soil, the top layer (0–30 cm) was mostly sand and rest was composed of aeolian parent material^52^. Based on the FAO soil taxonomy system^53^, the SW soil is classified as Histosol, the MD soil as Haplic Calcisol, while the SD was Arenosol.

For each soil type, six sampling plots of at least 50 × 50 m^2^ with a minimum distance of 100 m from each other were selected. At each sampling plot, three soil cores from the top 20 cm below the litter layer were collected using a 6.5 cm diameter steel push corer, and mixed into one approximately 1.5 kg homogenized composite sample. Visible plant roots and residues were removed before mixing. The corer was sterilized using 70% ethanol between samplings. Samples were stored in plastic bags on ice and divided into two portions. Sub-samples for physicochemical analyses and DNA extraction were stored at 4 °C and at −80 °C, respectively.

### Soil physicochemical properties

Soil pH and soil organic carbon (SOC) were determined using a soil-to-water ratio of 1:5 and dichromate oxidization method, respectively^54^. Soil gravimetric water content (WC) was determined by oven-drying at 105 °C for 48 h^54^. Soil total nitrogen (TN) and available nitrogen (AN), total phosphorus (TP) and available phosphorus (AP), total potassium (TK) and available potassium (AK) were determined by Kjeldahl digestion, alkaline hydrolysis diffusion method, and molybdenum blue method and flame photometry, respectively^54^.

### DNA extraction, amplification and sequencing

DNA was extracted from 0.75–0.91 g fresh weight soils (corresponding to 0.50 g dry weight) using the FastDNA Spin Kit for Soil (MP Biomedicals, Solon, OH, USA) following the manufacturer’s instructions. The quality of DNA was examined using the Nano-200 spectrophotometer (Aosheng, Hangzhou, China). DNA samples were stored at −20 °C.

PCR amplification was carried out using the primers 515F (5′-GTGCCAGCMGCCGCGGTAA-3′) and 806R (5′-GGACTACVSGGGTATCTAAT-3′) targeting the V4 hyper variable region in the bacterial and archaeal 16S rRNA gene^55^. The reverse primer was combined with the adapter and barcode sequences for multiplexing. Amplification was done in 50 μl-reaction volumes containing 3 U of TaKaRa Ex Taq HS (TaKARA Shuzo Co., Shiga, Japan), 5 mM dNTP Mixture (TaKARA), 2.0 mM MgCl_2_, 5 μl of 10× Ex Taq Buffer (TaKARA), 0.5 mM of each primer and 2.5 ng of soil DNA. The PCR procedure included an initial denaturation at 94 °C for 4 min, 30 cycles of 15S at 94 °C, 15S at 55 °C and 30S at 72 °C, and a final extension at 72 °C for 10 min.

PCR products were purified using PCR Clean-up Purification Kit (MP Biomedicals) and quantified using Qubit 2.0 fluorimeter (Invitrogen, Carlsbad, CA, USA). Purified amplicons were pooled in equimolar concentrations and sequenced using MiSeq Reagent Kit V2 for Illumina MiSeq. The raw sequence data were submitted to NCBI Sequence Read Archive (https://www.ncbi.nlm.nih.gov/sra/) with accession number SRS2127453.

### Bioinformatics analysis

Sequence reads were quality-filtered in QIIME (v1.9.1)^56^. Sequences with phred-quality score over 20 and longer than 300 bps were kept for downstream analyses while those could not be assembled were discarded. Chimeric sequences were removed using UCHIME against a reference alignment^55^. Sequences were clustered into operational taxonomic units (OTUs) at 97% using the UPARSE pipeline^57^. The RDP classifier was used to assign OTU representative sequences at 70% threshold^56^. For inter-group comparison, OTUs were randomly resampled at a depth of 12822 sequences. Singletons were eliminated during resampling, results in 35,014 OTUs. The phylogenetic tree was generated based on the representative sequences and used for UniFrac distance calculations.

### Statistical analysis

We applied principal component analysis (PCA) to assess differences in properties between soils. Analysis of similarity (ANOSIM) was used to test significant differences among abundances of taxa. The relative abundances of taxa at phylum and family levels were analyzed using R^58^. Two-way ANOVA was performed to test differences in the abundances of taxa at family level at P < 0.05. Principal coordinate analysis (PCoA) based on weighted and unweighted UniFrac distances were carried out in the R package vegan^59,60^. Distance based redundancy analysis (dbRDA) was performed to investigate factors driving community variation. Significance of factors was calculated by the permu test function in vegan over the dbRDA model using a maximum of 999 permutations. The goodness of fit for each edaphic factor was estimated by applying the envfit function in vegan (999 permutations). Mantel test was used to determine correlations between soil properties and microbial community composition.

The R package DESeq2 was used to analyze differential log_2_-fold transformed abundances at phylum and genus levels^61^. Differential abundance analysis was performed by fitting a generalized linear model with a negative binomial distribution to the normalized value for each type of taxa, and testing for differential abundance using a Wald test^61^. *P*-values were adjusted for multiple testing following the procedure of Benjamini and Hochberg^62^, and a false discovery rate (FDR) of 10% was selected to denote statistical significance^63^. Taxa with significant changes in abundance were defined as those that had differential abundance >1.0 and FDR-adjusted *P* < 0.1. Correlations between taxa with significant changes in abundance and edaphic features were determined using Spearman’s rank correlation.

### Network analysis

To reduce the complexity of the network, only OTUs with significant abundance variation among groups were considered for co-abundance network construction, which was then visualized in Cytoscape v3.3.0^64^. Firstly, a Spearman’s rank correlation coefficient matrix was calculated using “scipy” Python package for adjacency matrix construction^60^. To get robust links among nodes in the network, we further corrected all P-values using the Benjamini and Hochberg false discovery rate (FDR) as performed in “multtest” R package^58,65^. The cutoffs of correlation were determined as ±0.75 based on random theory-based methods^66^. The networks were generated using the “igraph” Python package based on cutoffs of correlation (FDR-P < 0.001)^67^. The network topological properties were calculated to evaluate the importance of each OTU (nodes). OTUs that were strongly correlated were screened using greedy modularity optimization algorithm^68^. To identify the primary driving force of modularity, we fit taxon abundance and edaphic factors to Bray-Curtis dissimilarities of modules to find the group of taxa and edaphic factors with highest correlation coefficient. The goodness of fit for each taxon and edaphic factor were estimated by the envfit function in vegan R package (999 permutations). Relations between the nodes of networks and soil properties were evaluated by Mantel test and partial Mantel test.

### Data accessibility

Sequencing data were submitted to NCBI Sequence Read Archive database (https://www.ncbi.nlm.nih.gov/sra/) with accession number SRS2127453. The OTU table is accessible in Dryad database with doi:10.5061/dryad.ks767.

## Electronic supplementary material

Supplementary Information

Dataset 1

Dataset 2
