# Supplementary material for: Degradation shaped bacterial and archaeal communities with predictable taxa and their association patterns in Zoige wetland at Tibet plateau
Source: Sci Rep. 2018 Mar 1;8:3884. doi: 10.1038/s41598-018-21874-0 (PMC5832768; doi:10.1038/s41598-018-21874-0)
Supplement: Supplementary file 1 — Supplementary Information [file 41598_2018_21874_MOESM1_ESM.docx]

**Supplementary materials for**

**Degradation shaped bacterial and archaeal communities with predictable taxa and their association patterns in Zoige wetland at Tibet plateau**

Yunfu Gu^1^, Yan Bai^1^, Quanju Xiang^1^, Xiumei Yu^1^, Ke Zhao^1^, Xiaoping Zhang^1^, Chaonan Li^2^, Songqing Liu^3^, & Qiang Chen^1*^

**Table S1** Sampling sites in Zoige Plateau wetland

| Sample code* | Sampling site | Longitude | Latitude | Altitude (m) | Soil temperature  (°C) |
| --- | --- | --- | --- | --- | --- |
| SW11 | Axi | 102°57′ 40.5″N | 33°48′32.8″E | 3427 | 5 |
| SW13 | Axi | 102°57′ 77.6″N | 33°43′61.9″E | 3430 | 5 |
| SW22 | Heihe | 102°48′ 48.2″N | 33°54′58.2″E | 3432 | 6 |
| SW23 | Heihe | 102°48′ 31.4″N | 33°53′23.3″E | 3433 | 6 |
| SW32 | Fenqu | 102°48′ 59.4″N | 33°55′21.2″E | 3436 | 5 |
| SW33 | Fenqu | 102°48′ 52.7″N | 33°55′34.1″E | 3427 | 5 |
| MD7 | Axi | 102°52′ 08.5″N | 33°47′37.4″E | 3465 | 4 |
| MD15 | Axi | 102°53′ 58.2″N | 33°48′25.1″E | 3452 | 4 |
| MD78 | Huahu | 102°56′ 94.9″N | 33°38′99.6″E | 3481 | 5 |
| MD62 | Huahu | 102°57′ 23.4″N | 33°38′99.6″E | 3481 | 5 |
| MD35 | Ruoergai | 103°02′ 08.6″N | 33°30′00.1″E | 3529 | 5 |
| MD14 | Ruoergai | 103°02′ 07.3″N | 33°29′54.5″E | 3514 | 4 |
| SD67 | Tangke | 102°33′ 39.8″N | 33°19′44.4″E | 3445 | 5 |
| SD68 | Tangke | 102°36′ 11.7″N | 33°16′20.4″E | 3536 | 5 |
| SD70 | Xiaman | 102°28′53.4″N | 33°41′38.4″E | 3492 | 5 |
| SD73 | Xiaman | 102°29′20.7″N | 33°42′07.8″E | 3522 | 5 |
| SD75 | Nenwa | 102°33′53.5″N | 33°52′16.9″E | 3528 | 4 |
| SD76 | Nenwa | 102°34′34.1″N | 33°52′07.5″E | 3471 | 5 |

* SW: swamp soil; MD: meadow soil; SD: sandy soil.

**Table S2** The network level topological features

| Node counts | Edge counts | Average degree | Average path length | Clustering coefficient | Cluster number | Modularity | Diameter | Degree assortativity | Density |
| --- | --- | --- | --- | --- | --- | --- | --- | --- | --- |
| 229 | 1893 | 16.53 | 2.78 | 0.49 | 27 | 0.39 | 7 | 0.18 | 0.073 |

**Fig. S1** The relative abundance of classes within phylum Proteobacteria.


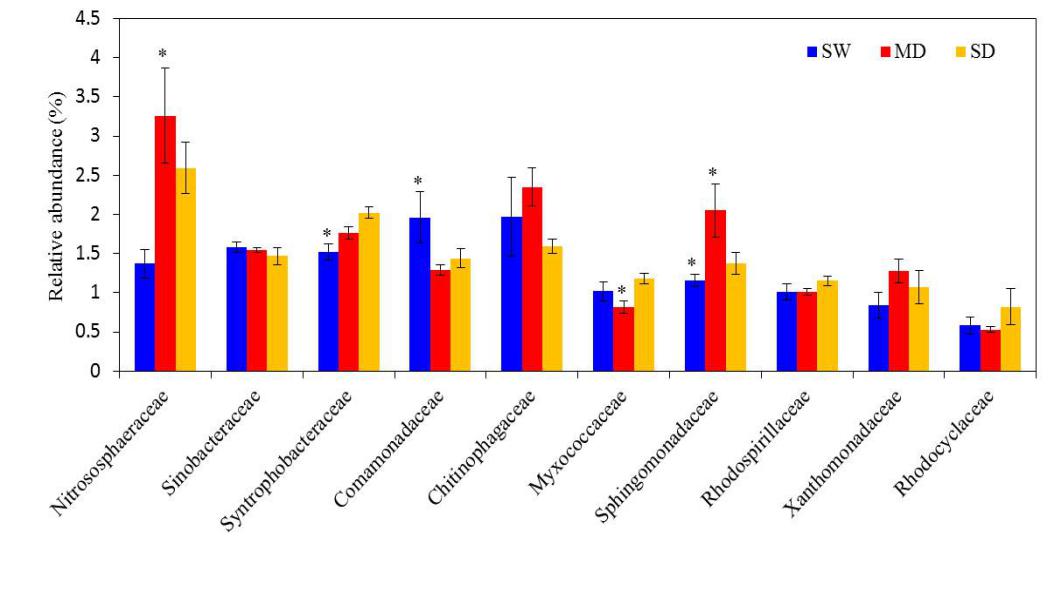


**Fig. S2** The relative abundance of top 10 families in each type of soil. SW: swamp soil; MD: meadow soil; SD: sandy soil.


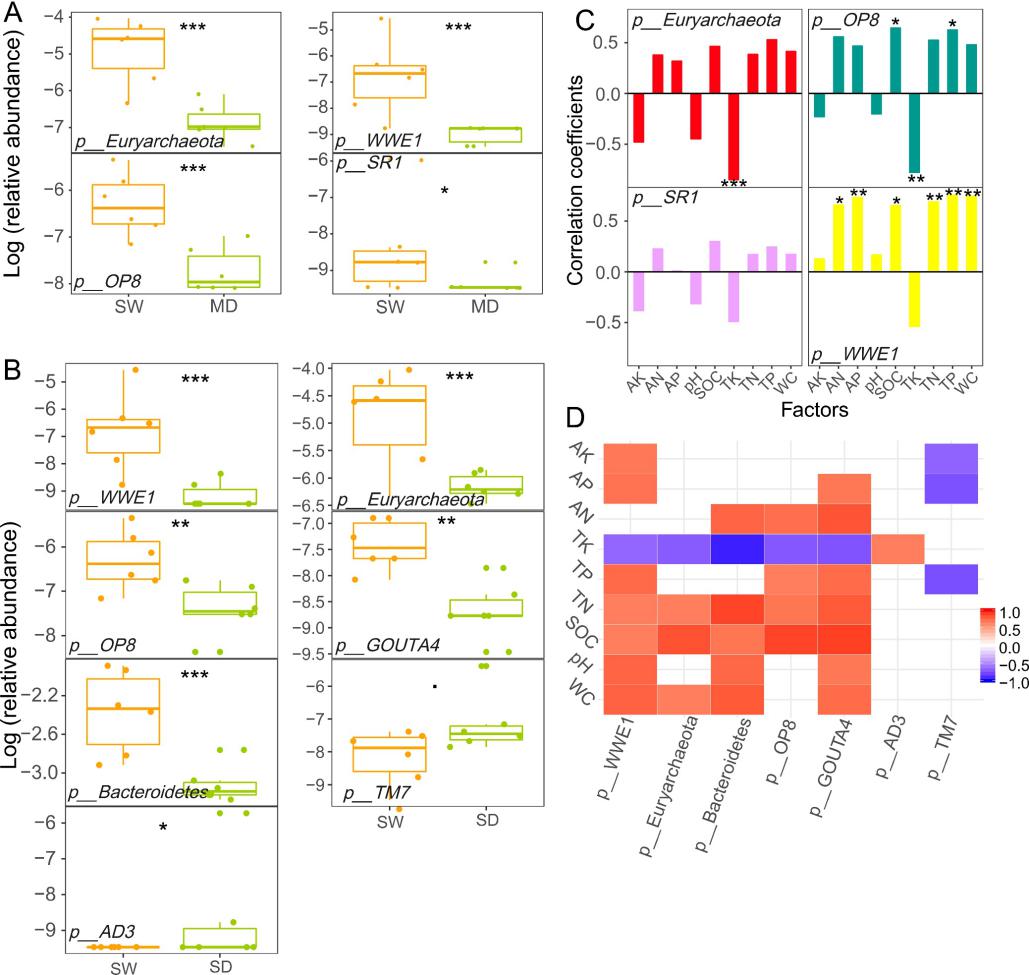


**Fig. S3** The boxplot of differential abundance phyla in swamp soil (SW), meadow soil (MD) and sandy soil (SD) (A and B), and associated potential determinants (C and D). Only correlation coefficients with P-values less than 0.05 were shown in sub-figure D. · *FDR-P* < 0.1, * *FDR-P* < 0.05, ** *FDR-P* < 0.01, *** *FDR-P* < 0.001. SOM, soil organic matter; WC, water content; TN, total nitrogen; TP, total phosphorus; TK, total potassium; AN, available nitrogen; AP, available phosphorus; AK, available potassium.

**A**


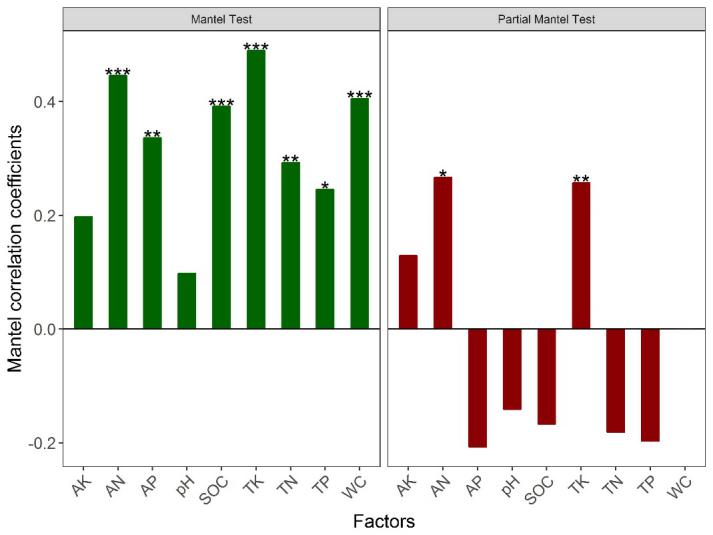

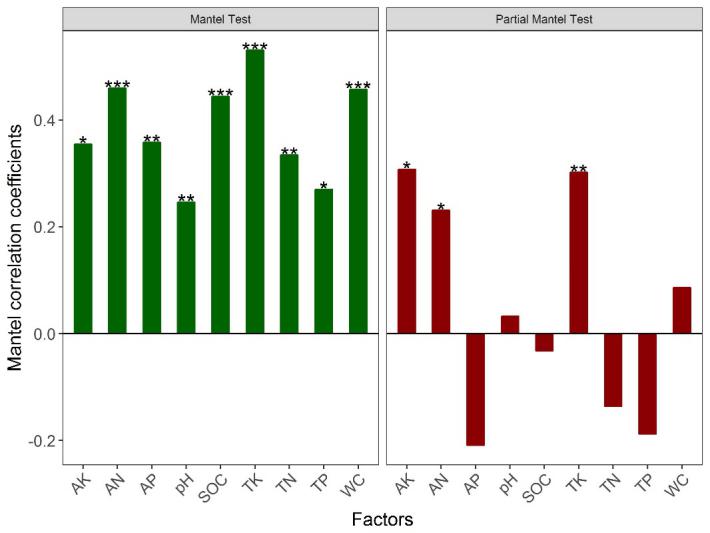


**B**

**Fig. S4** The mantel (darkgreen) and partial mantel test (darkred) based on weighted (A) and unweighted (B) UniFrac dissimilarity and spearman rank correlations. * *P* < 0.05, ** *P* < 0.01, *** *P* < 0.001.


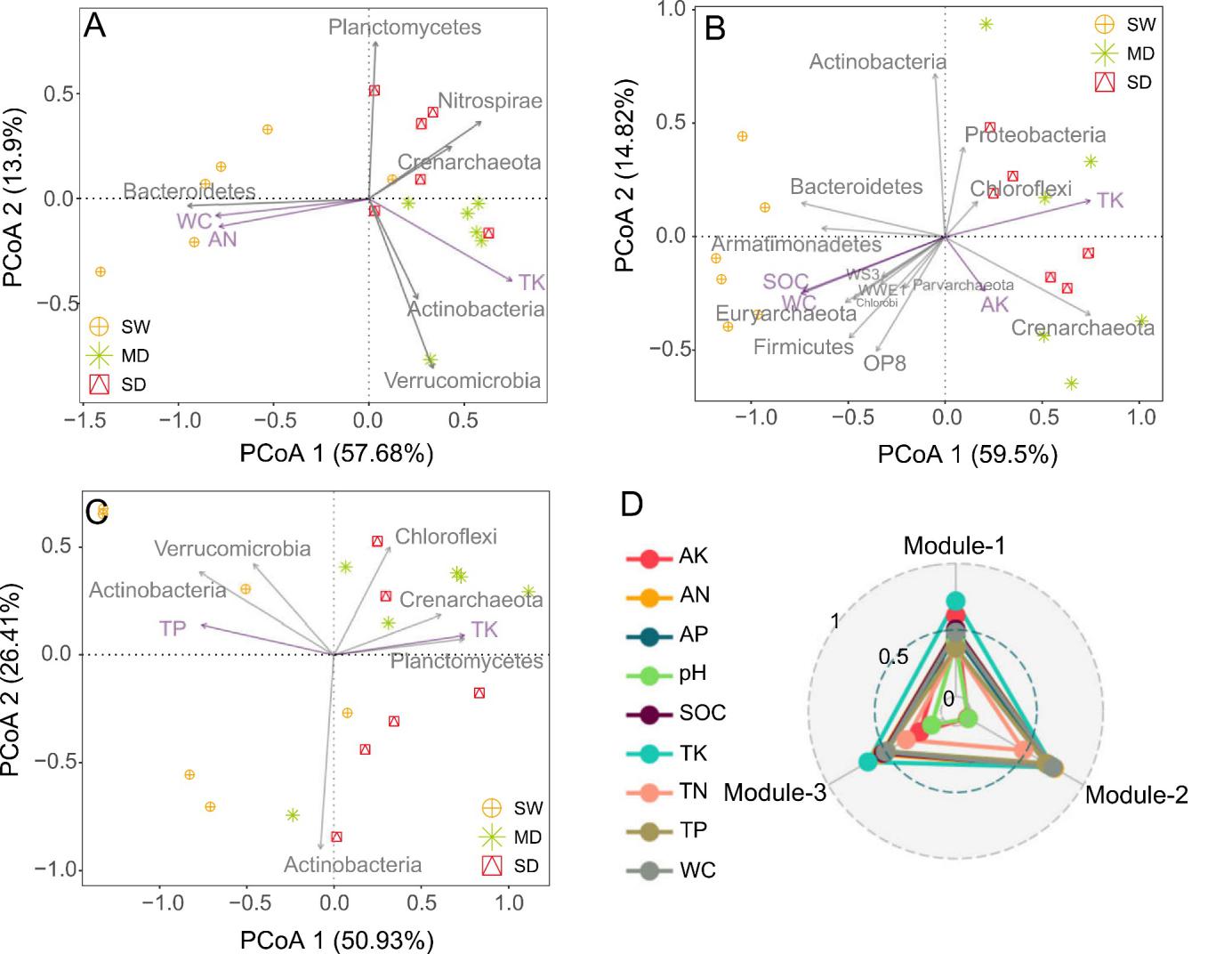


**Fig. S5** The relationships between taxon abundance, edaphic and potential functional microbiota (**A**: module1; **B**: module 2, and **C**: module 3), and the radar plot based on the Mantel test between edaphic and potential functional microbiota (D). See Table S1 for the soil codes and Table S2 for the soil edaphic factors.
